# Pirates of the haemoglobin

**DOI:** 10.15698/mic2022.04.775

**Published:** 2022-02-18

**Authors:** Daniel Akinbosede, Robert Chizea, Stephen A. Hare

**Affiliations:** 1 School of Life Sciences, University of Sussex, Falmer BN1 9QG, UK.

**Keywords:** nutritional immunity, haemoglobin, pathogenesis, virulence factors, TonB dependent transporters, NEAT proteins, ABC transporters

## Abstract

Not all treasure is silver and gold; for pathogenic bacteria, iron is the most precious and the most pillaged of metallic elements. Iron is essential for the survival and growth of all life; however free iron is scarce for bacteria inside human hosts. As a mechanism of defence, humans have evolved ways to store iron so as to render it inaccessible for invading pathogens, such as keeping the metal bound to iron-carrying proteins. For bacteria to survive within humans, they must therefore evolve counters to this defence to compete with these proteins for iron binding, or directly steal iron from them. The most populous form of iron in humans is haem: a functionally significant coordination complex that is central to oxygen transport and predominantly bound by haemoglobin. Haemoglobin is therefore the largest source of iron in humans and, as a result, bacterial pathogens in critical need of iron have evolved complex and creative ways to acquire haem from haemoglobin. Bacteria of all cell wall types have the ability to bind haemoglobin at their cell surface, to accept the haem from it and transport this to the cytoplasm for downstream uses. This review describes the systems employed by various pathogenic bacteria to utilise haemoglobin as an iron source within human hosts and discusses their contribution to virulence.

## INTRODUCTION

Haemoglobin (Hb) is probably the most famous protein in the world. Knowledge of its role in transporting oxygen around our bodies and the bright red colour it gives to blood is ubiquitous. Aside from this erythrocytic role, Hb has also been found in macrophages, alveolar epithelial cells and certain kidney cells, where it is proposed to function in reducing oxidative stress, and also in oligodendrocytes and some neurones, where it may act as an oxygen store [1, 2]. Another lesser-known feature of Hb, is its exploitation as a nutrient source by a substantial cohort of pathogens to support their survival, growth and virulence. Pirating essential elements from such a prominent source allows bacteria to circumvent the nutritional immunity employed as a defence mechanism by their hosts [3].

The utilisation of Hb and other serum proteins to promote survival and virulence is well documented for many pathogens, both eukaryotic and prokaryotic, which rely on host proteins for one nutrient in particular: iron [4, 5]. In the case of Hb, the iron comes in the form of haem. The use of this iron chelating compound ensures Hb is the largest iron source in the human body and is central to the ways in which some pathogens acquire iron within human hosts.

Structurally, Hb is a tetrameric polypeptide in erythrocytes comprising two alpha and two beta subunits, with each peptide chain containing one haem group [6]. The haem group is a prosthetic porphyrin ring and acts as an oxygen sequestering metal complex where iron is the central component. The release of Hb from erythrocytes during haemolysis increases the rate of oxidation of the Fe^2+^ centre, resulting in the change to met-haemoglobin (met-Hb). Met-Hb is the metalloprotein form in which the haem group iron is oxidised to the ferric (Fe^3+^) state and oxy-Hb refers to Hb bound to oxygen. The most relevant form of Hb for invading pathogens is likely to be met-Hb, because, unlike oxy-haemoglobin (oxy-Hb) which is mostly found in intact erythrocytes, met-Hb is more likely to be accessible to bacteria pathogens. The presence of four haem compounds per Hb molecule renders the protein both efficient at transporting oxygen and a very desirable target for invading pathogens with a requirement for an exogenous iron source.

A large majority (70%) of the iron in the human body is bound to haem. This haem is largely inaccessible to invading pathogens due to its secluded situation in the Hb subunit. Spontaneous or induced lysis of erythrocytes releases free Hb into the serum, which, as well as the conversion to met-Hb, typically sees the tetrameric protein break up into two dimers of one alpha and one beta subunit apiece. The dissociation of Hb implicates the rate at which haem is released into solution. Haem dissociates from monomeric and dimeric Hb subunits much quicker than from the tetramer [7, 8]. This is one of the reasons why the Hb dimers are quickly and tightly bound by haptoglobin (Hp), in the first stage of eventual recycling by macrophages and hepatocytes (**Table 1**) [9]. Hp is a liver synthesized plasma glycoprotein that is reported at an average concentration of 150 mg/mL in human adults [10]. Further degradation of Hb leads to a release of free haem, which is associated with cytotoxicity and oxidative damage. The potential toxicity of these events is mitigated in part by the proteins haemopexin and serum albumin in their role efficiently mopping up the free haem (**Table 1**)[11].

**TABLE 1.** **Concentrations and ligand affinities of serum proteins that play a role in scavenging haem and Hb.** A small amount of extraerythrocytic Hb is present in healthy individuals, but this is rapidly bound by haptoglobin. Any haem that is released from Hb will be bound with very high affinity by haemopexin, or by the abundant albumin. Together, these proteins protect from haem-mediated oxidative damage and restrict pathogens access to haem as an iron source.

**Haemoprotein**	Plasma concentration (mg/ml)*	**Ligand**	**Dissociation constant (K_D_) (M)**
Haemoglobin	0 - 0.4 [134]	Haem	10^−12^ - 10^−16^ [135]
Haptoglobin	0.3 - 2.1 [136]	Haemoglobin	<10^−15^ [137]
Serum albumin	34 - 54 ^†^	Haem	10^−8^ [138]
Haemopexin	1-2 [139]	Haem	<10^−13^ [140]

* Extraerythrocytic concentration range in healthy individuals.

† Normal concentration range for serum albumin tests.

As well as being the most abundant transition metal in living organisms and the environment, iron is a crucial metal for many biological processes. The vital necessity of iron is best demonstrated by its central role in the ubiquitous respiratory cytochromes, whose early discovery illustrates their pervasive importance to life [12, 13], but it is also a co-factor for many enzymes, such as class I ribonucleotide reductase, and aromatic amino acid hydroxylases [14, 15]. Iron's utility in biological process is likely due to its stability in ferric (Fe^3+^) and ferrous (Fe^2+^) states and considerable redox potential. However, free ferric iron generally forms highly insoluble oxides and can be described as biologically inoperative in most cases. This great redox potential does come with some negatives, as iron ions can catalyse the production of highly reactive hydroxide radicals. These hydroxide radicals are involved in several destructive processes when they react with biomolecules such as DNA and lipids. To mitigate this potential toxicity, a carefully balanced range of regulatory processes prevent hosts from reaching toxic concentration levels of iron. These regulatory processes are broadly referred to as iron homeostasis and include uptake, storage and excretion [16].

During infection, pathogens are entirely reliant on nutrients sourced from their host. So much so, that mammalian hosts, including humans, have developed several mechanisms to sequester trace minerals to restrict the growth of infecting microorganisms. Circulating iron levels are further depleted upon infection and neutrophil granules of neutrophils contain high levels of the iron-sequestering protein lactoferrin to release at sites of inflammation. This process is referred to as nutritional immunity [17, 18]. Both iron homeostasis and nutritional immunity create significant barriers for invading pathogens. Consistent with the Red Queen hypothesis, which suggests that direct competition is a driving force of evolution, pathogens have evolved an array of nutrient piracy mechanisms to counter nutritional immunity and to scavenge for free nutrients and even acquire nutrients from host proteins. In this review we will outline some of the systems deployed by bacterial pathogens to circumvent host nutritional immunity, specifically those that utilise Hb as an iron source. Other reviews have focused on the whole haem uptake process in Gram negative bacteria [19], on the system employed by *Staphylococcus aureus* and other gram positive organisms [20–25] on the structural aspects of the haemoprotein receptors [26], or given a brief overview of bacteria-haemoglobin interactions [27]. Here we take a broad approach in covering diverse bacterial pathogens but with a narrow focus on direct interactions with Hb and further discussion of the relevance of these interactions to pathogenesis. The intricacies of the diverse systems described here are unique, but the broader picture is familiar across the board: Hb is bound at the cell surface, haem is released and transported across the cell wall, haem is imported into the cell via an ABC transporter then degraded to release free iron for oxidative use by the cell.

## USE OF HAEMOGLOBIN BY GRAM POSITIVE BACTERIA

The single lipid bilayer and thick peptidoglycan layer of Gram positive bacteria serve both structural and functional purposes. In the business of pirating Hb for haem, it presents unique challenges. These challenges are bypassed using transmembrane ATP binding cassette (ABC) transporters, which are typically made up of a haem binding protein, permease and ATPase. These proteins along with a variety of extracellular and surface attached proteins to reach beyond the cell wall for direct contact with Hb. In this section we explore the details of and make comparisons between examples of these systems.

### Corynebacterium diphtheriae

*Corynebacterium diphtheriae* is a Gram positive, nonmotile human pathogen. It is the causative agent of the disease diphtheria and the production of the Diphtheria toxin, which is one of the most extensively studied bacteria toxins in history and is iron-regulated [28, 29]. *C. diphtheriae* infects by colonising the mucosal surfaces of the pharynx and the toxin is responsible for symptoms such as nausea, swollen glands and difficulty breathing and swallowing.

Among Gram positive bacteria, *C. diphtheriae* was the first to have a haem utilising protein characterised. HmuO was discovered via its homology with eukaryotic haem oxygenases and its ability to restore haem utilisation to *C. diphtheriae* mutants previously unable to use haem and Hb as iron sources [30]. Haem oxygenase proteins, such as HmuO, are essential for accessing the iron embedded within haem once in the cytoplasm and are widespread among Hb-utilising pathogens, however this review will focus mainly on the Hb binding events at the cell surface.

Haem acquisition in *C. diphtheriae* also depends on a haem specific ABC transporter system named HmuTUV [31]. HmuTUV is able to transport haem acquired from a wide range of host haemoproteins as substrate, including Hb, the Hb:Hp complex and myoglobin [32]. HmuTUV is made up of three proteins; HmuT which binds haem, HmuU which functions as a permease and HmuV the ATPase. These proteins work in sequence to bring haem into the cytoplasm.

**Figure 1:**
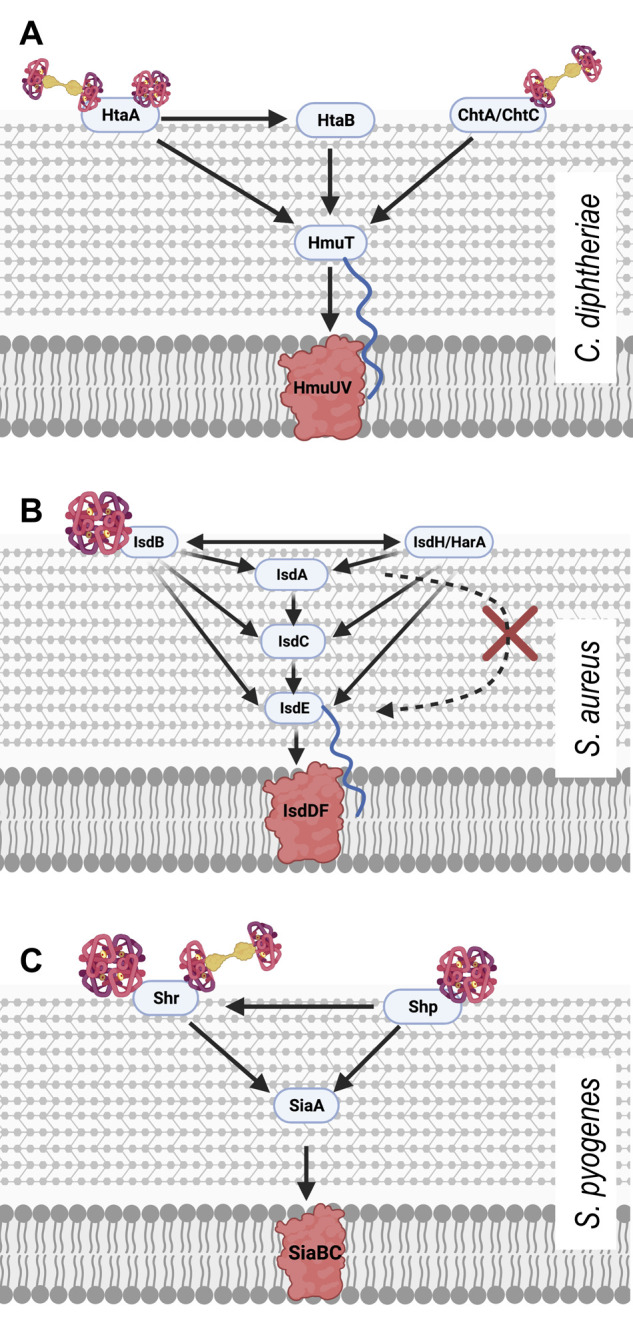
FIGURE 1: Schematic models of haem piracy from haemoglobin by the different Gram positive pathogens described herein. **(A)** In *C. diphtheriae*, two groups of surface exposed proteins bind Hb and Hb:Hp. HtaA is able to bind Hb and the Hb: Hp complex while HtaB is responsible for binding free haem, meanwhile ChtA/ChtC proteins bind directly only to the Hb: Hp complex. Both groups of proteins transfer haem to HmuT, the lipoprotein component of the ABC transporter for import through the lipid bilayer. **(B)** In *S. aureus*, surface exposed proteins IsdB or HarA/IsdH bind haemoglobin at the cell surface and strip it's haem. The haem can them be passed to any of the proteins IsA, IsdC and IsdE and unidirectional transfer from IsaA – IsdC – IsdE leads to transport to the cytoplasm through the IsdDEF ABC transporter for transport into the cytoplasm. **(C)** At the surface of *S. pyogenes*, Shp extracts haem from Hb and Shr is able to obtain haem from both Hb and the Hb:Hp complex. These proteins transfer haem to lipoprotein SiaA for transport into the cell via SiaABC.

The *hmuTUV* operon also includes *htaA* and neighbours the *htaB* gene both of which encode surface exposed haem binding proteins, HtaA and HtaB, of which HtaA is solely responsible for taking haem from haemoglobin, and HtaB binds free haem (**Figure 1A**) [33]. HtaA has been shown to possess two functionally important domains, CR1 and CR2, both of which, when recombinantly expressed with glutathione S-transferase tags, were shown to bind haem though UV-visual spectroscopy. This work, however, did not demonstrate the ability of each domain to bind Hb itself. Using Hb derived enzyme-linked immunosorbent (ELISA) assays, CR2 was shown to have a much higher affinity for Hb than CR1. Interestingly, it was also demonstrated through competitive binding studies that HtaA preferentially binds to Hb that is carrying haem. Alignment studies, followed by mutagenesis studies revealed two residues that are essential for CR2 domain binding to Hb; Y361 and H412. Beyond just binding Hb, *in vivo* assays involving the deletion of the Y361 residue revealed that it is essential for Hb utilisation via HtaA. These data overwhelmingly made the case for the importance of HtaA in the utilisation of Hb but it is still important to note that HtaA is able to transfer haem to HtaB [34], presumably to make the process of transport to the cell membrane more efficient.

The importance of HtaA and HtaB to haem acquisition in *C. diphtheriae* is now well documented. Additionally, the identification and characterisation of the CR domains of HtaA has been a useful tool in identifying other Hb binding proteins that are part of the *C. diphtheriae* haem acquisition system, such as ChtA, ChtB and ChtC. ChtA/ChtC are surface anchored proteins previously shown to have structural similarities to HtaA. ChtB meanwhile, although some evidence suggests that it binds Hb using a CR domain, shows high levels of sequence similarities with the haem-binding HtaB. In fact, both ChtB and HtaB have been linked to haem transport due to significantly decreased haem use in ChtB/HtaB double mutants [35]. In 2015, Allen and Schmitt reported that double deletion mutants of ChtA and ChtC rendered *C. diphtheriae* incapable of acquiring haem from the Hb:Hp complex, although single deletion mutants of either protein resulted in wild type haem acquisition capabilities, suggesting redundancy between the proteins [36]. HtaA and ChtaA/ChtC have a great deal in common. They have high sequence similarity and are similar in cellular location, size and structure. Their shared CR domains also allow all three proteins to bind both haem and Hb. These proteins however are not without their differences. Whilst HtaA is able to play a part in haem acquisition from Hb, Hb:Hp and myoglobin, ChtA/ChtC is only involved in haem acquisition from the Hb:Hp complex, suggesting these proteins must work in tandem with HtaA in Hb:Hp utilisation (**Figure 1A**) [36]. In addition to the proteins possessing CR domains, *C. diphtheriae* is also able to bind the Hb:Hp complex using HbpA, a recently identified surface exposed protein that is both secreted and anchored to the membrane. HbpA was identified due to proximity to a haem responsive two-component signal transduction system and has been shown to be of significant importance in *C. diphtheriae* acquisition of haem from the Hb:Hp complex. Indeed, HbpA deletion mutants in iron depleted media showed reduced ability to use the Hb:Hp complex as an iron source, while showing no difference in the use of Hb or haem. [37]

In summary, HtaA, HtaB, ChtA and ChtC are surface exposed proteins of *C. diphtheriae* and are able to bind host haemoproteins, starting the cascade of haem-passing interactions that follow; for example, HtaA to HtaB to HmuT then onto HmuUV for transmembrane transportation. The ability to use Hb as an iron source is not unique within the genus to *C. diphtheriae*. HtaA proteins can also be found in the horse pathogen *Corynebacterium pseudotuberculosis* [38] and the rarer cause of diphtheria in humans, *Corynebacterium ulcerans* [39].

### Staphylococcus aureus

*Staphylococcus aureus* is a Gram positive bacterium found on approximately 25% of all humans as part of the skin or nasal flora [40]. *S. aureus* is also an opportunistic pathogen that can cause infection upon breaking through the skin barrier to cause a multitude of skin and blood infections. These include abscesses, boils, bacteraemia, endocarditis, pneumonia, empyema, osteomyelitis, toxin-mediated food poisoning, scalded skin syndrome, and toxic shock syndrome [41]. *S. aureus* has an abundance of virulence factors it can use to cause infection upon invasion. It produces a variety of proteins and enzymes to aid in its survival in multiple niches.

Like most bacteria, *S. aureus* has developed systems for iron piracy in order to facilitate its survival in iron deficient environments. One of these systems comprises a series of iron-regulated surface determinant (Isd) proteins that work together to bind Hb, extract haem and transport it to the cell membrane for transport into the cytoplasm [20, 23, 25, 42].

The Isd system of *S. aureus* is made up of four proteins which are anchored to the cell wall and work in canon - IsdA, IsdB, IsdC, IsdH (also known as HarA). Isd proteins found in the cell wall all possess a ‘NEAr Transporter' otherwise known as NEAT domain [22]. NEAT domains are capable of binding haem and transferring it through a cascade of nearby Isd proteins toward the cell membrane. The Isd system also possesses another five proteins, IsdE, IsdF are embedded in the cell membrane and make up the ABC transporter essential for transporting haem into the cytoplasm, similar to HmuTUV of *C. diphtheriae*. IsdG and IsdI are haem oxygenases (similar to HmuO of *C. diphtheriae*) required for gaining access to the iron stored in the transported haem [24, 43]. IsdD, though previously suspected to interact with IsdE and IsdF in the ABC transporter cassette, currently has no known function [21].

Of the haem-binding Isd proteins, IsdB was originally shown to be critical for binding Hb directly to the bacterial cell surface and for extricating the haem from it (**Figure 1B**). A 2008 study by Muryoi *et al.* using magnetic circular dichroism spectra and electrospray ionization mass spectrometry data demonstrated that haem can be transferred from NEAT domains on IsdA, B or C to IsdE [43]. It was reported that these transfer events happen in a unidirectional manner with IsdB and IsdH as potential starting points. In the IsdB pathway, IsdB is able to pass haem onto IsdA which passes it on to IsdC. The other pathway differs in that the haem can be sourced from intact Hb or the Hb:Hp complex, both of which bind the first NEAT domain of IsdH. The haem is released from the IsdH-bound Hb and is initially bound by third NEAT domain of IsdH [44], which then passes haem on to IsdA, joining the same pathway as above. In both cases the haem bound to IsdC is then passed on to IsdE - the haem binding protein of the Isd ABC transporter, responsible for transporting haem into the cytoplasm of the cell. In the scope of the experiments by Muryoi *et al.*, it was reported that these haem transfers were unidirectional, where IsdE could only accept haem directly from IsdC and not IsdA. A second study confirmed the unidirectional transfer specifically using haem acquired from met-Hb; showing that IsdB strips heam from metHb via NEAT B2 and then transfers it to the membrane through the IsdA – IsdC – IsdE canon [45]. The lack of direct transfer between IsdA and IsdE, positions IsdC as an essential component of the haem transfer pathway. Interestingly, Pilpa *et al.* in 2009 also observed a direct but less-efficient acquisition of haem from Hb by IsdC *in vitro*, suggesting that there may be additional flexibility in the system albeit the limited accessibility of IsdC would likely hamper this transfer further *in vivo* [44]. In another interesting observation of the flexibility of the system, the two initial haem capturing proteins IsdH and IsdB could transfer haem bidirectionally exclusively amongst themselves (**Figure 1B**) [43]. This is probably because both proteins are very closely related, and both possess at least two NEAT domains which are actively involved in the binding to Hb and stripping of haem as concluded from crystallography analysis [46]. The transfer of haem between NEAT domains is highly specific and kinetics studies have shown that the transfer from IsdA to IsdC and IsdC to IsdE respectively happens >70,000 times faster and ∼4-10 times faster than spontaneous release of haem into solvent, allowing for their classification as specific haem chaperones [22, 45, 47].

IsdB and its Hb utilising capabilities have also been identified as an important aspect of *S. aureus* virulence. *isdB* deletion mutants displayed a reduction in virulence in a murine model of abscess formation. This same reduction was not observed with *isdH* mutants, suggesting that IsdB facilitated haem piracy directly from Hb is crucial in full *S. aureus* virulence [48]. By contrast, functional homologues of these NEAT proteins are not discernible in less virulent species of the *Staphylococcus* genus such as *Staphylococcus epidermidis* and *Staphylococcus saprophyticus*.

### Streptococcus pyogenes

*Streptococcus pyogenes*, also known as group A streptococcus, is a species of Gram positive, nonmotile cocci bacterium that is a member of the normal human nasopharyngeal flora. Immune evasion by *S. pyogenes* can lead to pathogenesis and cause local infections such as pharyngitis and scarlet fever, skin infections such as pyoderma and erysipelas, deeper soft tissue infections such as cellulitis and necrotising fasciitis (streptococcal gangrene), or systemic infections including streptococcal toxic shock syndrome (reviewed in [49]).

As a human pathogen, *S. pyogenes* is reliant on the human body for iron sources. *S. pyogenes* is able to bind and utilise free haem as well as several haem carrying host proteins as iron sources, including Hb, myoglobin, and the Hb:Hp complex. The inability to use transferrin, lactoferrin or other iron carrying host proteins as iron sources makes the haem utilisation of *S. pyogenes* even more significant. A 2003 study by Bates *et al.* showed that haem and haemoprotein utilisation in *S. pyogenes* occurs via an ABC transporter, named SiaABC (streptococcal iron acquisition) also referred to as HtsABC [50]. They demonstrated that nine clinical isolates of *S. pyogenes* bind Hb at the cell surface in a cell concentration-dependent manner by probing for Hb using a streptavidin alkaline phosphatase reporter system. The *siaABC* transporter genes are contained on an iron-regulated operon of ten genes, the first of which also encodes for a protein that is proposed to bind host haemoproteins. The researchers named this protein Shr (streptococcal haemoprotein receptor). Shr was shown to bind Hb, myoglobin and the Hb:Hp complex (but not Hp alone) through western blot analysis [50] (**Figure 1C**). Shr deletion mutants exhibited decreased levels of growth in human blood and diminished virulence in zebrafish infection models demonstrating that the protein is paramount to *S. pyogenes* virulence [51]. Shr binds to Hb using two unique Hb interacting domains (HIDs), named HID1 and HID2. Like the Hb-binding IsdB from *S. aureus*, Shr also possesses two NEAT domains that work together to accept haem from Hb. In the case of Shr, they are called NEAT 1 and NEAT 2 and are located in the N -terminal region of the protein [52]. Another product of this operon, Shp (Surface exposed haem binding protein) accepts haem from Shr (**Figure 1C**). Although widely regarded as a distant relative and not a direct member of the NEAT protein family, Shp is able to bind haem using structural similarities with NEAT family proteins [53]. Once in possession of haem, Shp is able to rapidly transfer haem to SiaA, the haem binding lipoprotein of the siaABC transporter [51]. Recombinant production of the ABC transporter component SiaA allowed for solid-phase binding assays which demonstrated that SiaA could bind Hb *in vitro*. Although the SiaA transporter system allows *S. pyogenes* to utilise several host haemoproteins, the same experiment showed that SiaA could not bind myoglobin or the Hb:Hp complex. The other components of *siaABC* encode a membrane permease (SiaB) and an ATPase (SiaC) [50].

In addition to studies of *shr* mutants, *siaA* has also been linked with virulence. Infection of mice with *siaA* deletion mutants resulted in significantly increased survival rate, reduced skin lesion size, and reduced systemic dissemination when compared to the wild type. The reinstatement of *siaA* restored *S. pyogenes* wild type capabilities suggesting that its ability to utilise haem is an important component for *S. pyogenes* infection [54].

### Bacillus anthracis

The detailed examples above by no means constitute an exhaustive list of Gram positive bacterial pathogens that are able to use Hb as an iron source. The conserved nature of the haem- and sometimes Hb-binding NEAT domains amongst Gram positive organisms suggest the ability to pirate haem from Hb is widely distributed. *Bacillus anthracis* is the pathogen responsible for the infamous infectious disease anthrax. Anthrax infections are typically started by *B. anthracis* spores gaining access to the host via breaks in the skin or entry through the mucosa. Although the symptoms of infection differ slightly between inhalation and gastrointestinal sites, fever, nausea, vomiting and stomach pains are universal [55]. *B. anthracis* is able to acquire haem from Hb by secreting extracellular haemophores IsdX1/X2 which are reported via ELISA style assays to bind Hb and accept haem. The haem-saturated haemophores then make their way to the cell wall where the haem is rapidly passed to surface exposed NEAT domain protein IsdC. From IsdC, haem is transferred via the transmembrane ABC transporter into the cytoplasm [56]. Although *B. anthracis* IsdC is crucial in this pathway of haem acquisition from Hb*,* IsdC deletion mutants did not lose their virulence in a guinea pig infection model [57]. In 2012, Balderas *et al.* used NEAT domain homology searches to demonstrate that the cell attached protein Hal (Haem-acquisition leucine-rich repeat) possessed a NEAT domain and was able to bind to Hb and accept its haem. Deletion mutants of Hal showed reduce growth in media in which either Hb or haem were the sole iron source [58].

Interestingly, another human pathogen in the *Bacillus* genera, *Bacillus cereus,* is able to use Hb as an iron source and possesses NEAT domain homologs but is incapable of using other iron-carrying host proteins such as transferrin or lactoferrin [59, 60]. *B. cereus* is a pathogen most closely associated with food poisoning but is now also linked with serious and potentially fatal non-gastrointestinal-tract infections [61]. There is no evidence that the non-pathogenic *Bacillus subtills* possesses the NEAT domain proteins needed to use Hb as an iron source.

### Listeria monocytogenes

Another pathogen deserving of an honourable mention is the foodborne pathogen *Listeria monocytogenes*. Infections with this pathogen can be responsible for gastroenteritis, meningitis, encephalitis and maternofoetal infections [62]. *L. monocytogenes* is able to pirate haem from Hb via Hbp2, a protein that is reported to exist both as a secreted haemophore as well as a surface attached protein. Hbp2 has three NEAT domains that facilitate its binding to both haem and Hb. Secreted Hbp2 is able to bind Hb and accept its haem before transferring it to surface-bound Hbp2 or to the closely related Hbp1. Once in possession of haem, surface bound Hbp1 or Hbp2 begin a cascade of haem transfers similar to that of the *S. aureus* Isd system toward the cell membrane, where the haem is transported into the cell by ABC transporter HupDGC. Interestingly, surface bound Hbp2 is also able to bind Hb directly [63, 64].

### Clostridium perfringens

*Clostridium perfringens* is another mostly foodborne pathogen that can utilise Hb as an iron source. *C. perfringens* infections can result in gas gangrene, food poisoning, non-foodborne diarrhoea and enterocolitis [65]. The mechanics of Hb piracy by *C. perfringens* are still poorly understood but the *C. perfringens* strain JIR325 is able to survive on Hb as a sole iron source [66]. Two haem-binding, NEAT domain-possessing proteins, ChtD and ChtE can be found on the cell surface and were initially suspected to be prime candidates for Hb utilisation capabilities. Both single and double mutants of the proteins did not strip *C. perfringens* of its ability to grow on media in which Hb was the sole iron source, although the mutants did exhibit reduced virulence in mouse myonecrosis models. Downstream of the ChtD and ChtE genes, a *C. perfringens* haem ABC transporter has been identified; ChtA - a haem binding lipoprotein, ChtB – a permease and ChtC1 an ATP binding protein, all of which are needed for transport into the cytoplasm [66].

## USE OF HAEMOGLOBIN BY GRAM NEGATIVE BACTERIA

**Figure 2:**
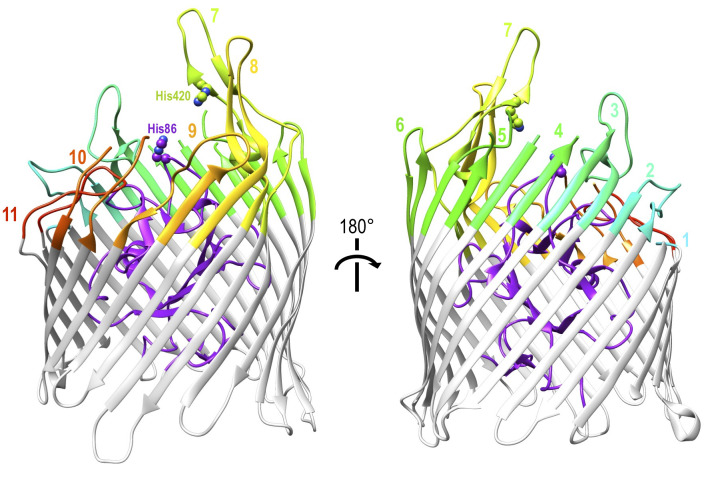
FIGURE 2: Cartoon representation of a TonB dependent transporter involved in Hb piracy. This is ShuA from *S. dysenterieae,* pdb id: 3fhh. The N-terminal plug domain is coloured purple, the membrane-enclosed beta barrel in light grey and the extracellular loops involved in Hb recognition and haem extraction are rainbow coloured from cyan for loop one to red for loop 11. Two conserved histidine residues proposed to bind to haem are labelled and shown as balls.

The cell wall structure and outer membrane of Gram negative bacteria present additional challenges to nutrient uptake. Because of the similarity of these challenges among Gram negative bacteria, haem uptake from Hb utilises the well-conserved TonB-dependent system that is fundamental to all active iron uptake in these bacteria [67]. They must not only bind to Hb but obtain the iron-containing haem from it, transport it across the outer membrane and then across the inner membrane into the cytoplasm. TonB-dependent transporters (TBDTs) are made up of a 22-strand transmembrane β-barrel protein with a globular N-terminal plug domain and eleven extracellular loops to facilitate substrate binding (**Figure 2**). The system is powered utilising energy derived from the proton motive force transmitted from the TonB-ExbB-ExbD complex located in the inner membrane [68]. Using the ferrichrome TBDT FhuA from *Escherichia coli* as a structural template, sequence analysis demonstrated that at least 50% of residues were conserved amongst twelve unique TBDTs. The regions of highest sequence conservation were the N-terminal plug domain and the β-strands of the barrel, indicating the core structures of the proteins are maintained, while none of the conserved residues were found on the extracellular loops reported to facilitate substrate specific binding [68]. Although they can be found in the outer membrane of almost all Gram negative bacteria, TBDTs are substrate specific transporters and the conservation of the TBDT system itself does not limit the range of possible substrates, which are diverse in structure and size and include iron-carrying siderophores, carbohydrates, lignin derived aromatic compounds or indeed host proteins such as Hb [69, 70]. This section outlines the ways in which TBDTs are used by a range of pathogenic Gram negative bacteria to acquire haem from Hb.

### Shigella dysenteriae

*Shigella dysenteriae* is a Gram negative, facultative anaerobic bacterium. It is a highly virulent pathogen and is usually transmitted by contaminated food and water and invades the host via epithelial cells of the intestinal mucosa [71, 72]. *S. dysenteriae* infections are associated with poor hygiene conditions resulting in a condition called shigellosis. Shigellosis commonly presents with symptoms such as diarrhoea that may be bloody, high fever and stomach cramps [73].

**Figure 3:**
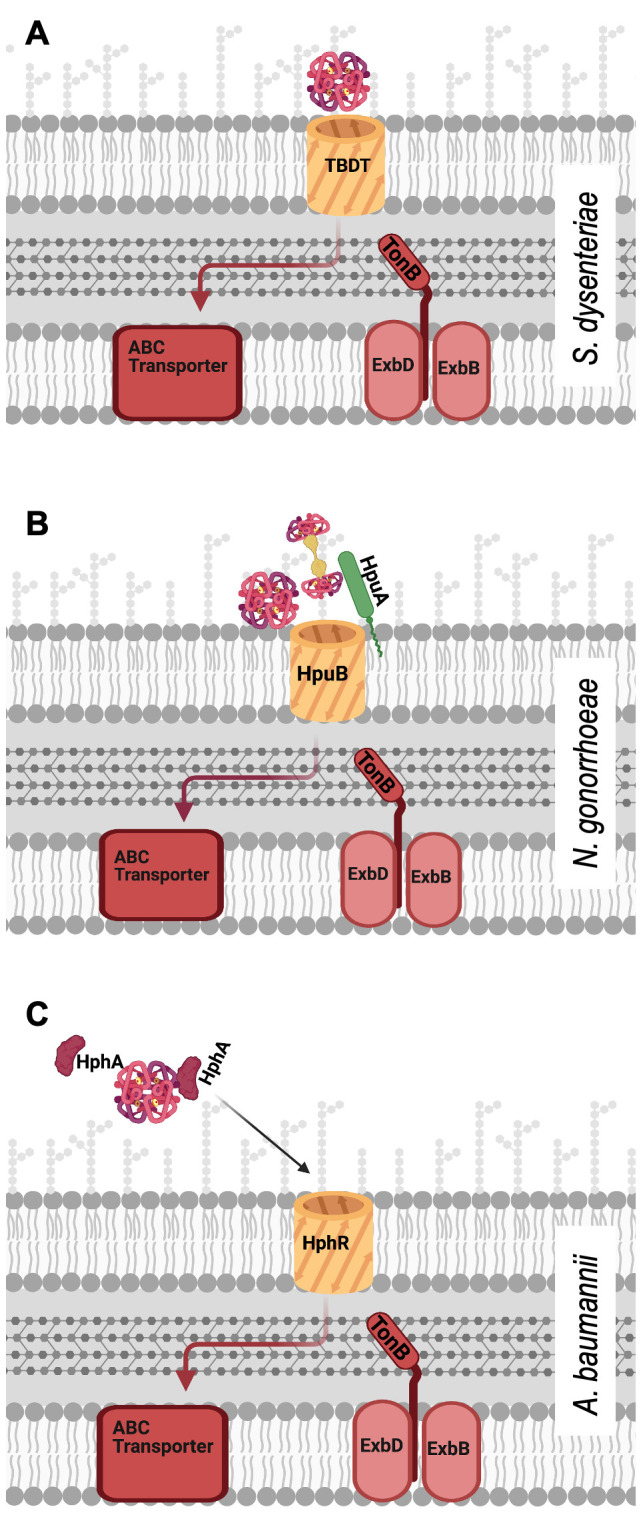
FIGURE 3: Schematic models of haem piracy from haemoglobin by the different Gram negative pathogens described herein. **(A)** Some pathogens, such as *S. dysenteriae*, employ monopartite TonB-dependent transporters (TBDT). These β-barrel outer membrane proteins (ShuA in the case of *S. dysenteriae*), enable active transport of haem from the host haemoprotein into the periplasm from where the haem is transported into the cytoplasm by a haem specific ABC transporter. **(B)** Some TBDTs, such as the gonococcal HpuB, work in conjunction with an associated surface exposed lipoprotein (HpuA) to extract haem from Hb or Hb:Hp and transport it into the periplasm. **(C)** Other TBDT systems, such as the A. baumannii HphR/HphA are a different kind of bipartite system. HphA is a secreted protein that is reported to bind Hb and brings its haem to the membrane for transport into the periplasm by HphR.

Key to the virulence and pathogenesis of *S. dysenteriae* is the acquisition of iron, in part through the Hb-binding TBDT ShuA. Although it is important to note that *S. dysenteriae* does also produce siderophores and has TBDTs that are specific to their uptake. *S. dysenteriae* colonises the intestinal mucosa where there is an abundance of haem due to both dietary haem intake and haemolytic damage caused by haemolytic *Shigella* toxins [74]. ShuA binds to Hb, accepts haem from it, then transports it across the outer membrane into the periplasm (**Figure 3A**). *In vitro*, ShuA extracts haem from met-Hb ∼2000 times faster than that from oxy-Hb [74]. This suggests met-Hb is the physiological substrate of ShuA *in vivo*, although the fact that met-Hb spontaneously releases haem faster than oxyHb may have influenced the experiment [7]. Indeed, met-Hb is the most likely available source of haem following *S. dysenteriae*-induced dysentery in the low oxygen environment of the gut.

Unique among Hb-binding TBDT homologues, the crystal structure of ShuA has been resolved (**Figure 2**) [75]. The structure conforms to the expected 22-stranded β-barrel with an N-terminal plug domain. The importance of two conserved histidine residues, His86 and His420, which were previously identified as crucial for the extraction of haem from Hb by observing loss of function in respective double mutants [74], was also indicated by the crystal structure of haemophore TBDT, HasR from *Serratia marcescens*, where a haem molecule was observed coordinated between two corresponding residues [76]. Hb binding and haem transfer on to ShuA are dependent on the conserved residues His86 and His420 with other interactions also documented to play a role in haem piracy [75]. A structural explanation of the way ShuA is able to remove haem from Hb is still elusive, but the crystal structure obtained from ShuA is now a crucial tool in modelling and understanding how other Hb-specific TBDTs work.

The role of ShuA during infection is still unclear but disrupting the *shuA* gene resulted in a strain that was incapable of using haem as an iron source, suggesting that ShuA is essential for haem utilisation in *S. dysenteriae* [77]. In addition, very similar TBDTs can be found in the genomes of other pathogenic *Shigella* species: *Shigella sonnei* and *Shigella flexneri.*

### Neisseria

*Neisseria* are a genus of mostly non-pathogenic, Gram negative and aerobic bacteria that are a significant part of the human microbiome [78]. The genus comprises at least eleven species that colonise humans, two of which are pathogenic. Whilst their respective infections present very different symptoms, *Neisseria meningitidis* and *Neisseria gonorrhoeae* are closely related, with DNA primary sequence homology of between 80 and 90% [79]. *N. meningitidis* is able to colonise the nasopharynx and can lead to rapidly fatal symptoms such as high fever, inflammation of the meninges and sepsis if not promptly treated with appropriate antibiotics [80]. *N. gonorrhoeae* is the pathogen responsible for the sexually transmitted infection gonorrhoea and infects via the mucosal membranes of the reproductive tract. Where present, symptoms of gonorrhoea include discharge from infected genitalia, however, the infection is often asymptomatic, particularly in female patients. If undetected and untreated, *N. gonorrhoeae* infections may ascend the reproductive tract and develop into pelvic inflammatory disease and potentially cause infertility. There is currently growing concern about the rise of antibiotic resistance in *N. gonorrhoeae* [81].

*N. gonorrhoeae* and *N. meningitidis* have no reservoir other than humans and are thus adepted at utilising host resources for their survival and pathogenesis. Pathogenic *Neisseria* obtain their iron utilising a wide range of sources, from hijacking iron chelating siderophores made by other bacteria to host iron-carrying proteins such as transferrin. Although pathogenic Neisseria can survive solely on iron acquired from transferrin, they do pirate the metal from Hb in the form of haem via the Hb specific TDBT systems HpuAB and, in the case of *N. meningitidis,* HmbR.

HpuAB and HmbR are both TBDTs, powered by the highly conserved TonB-ExbB-ExbD complex and are both subject to high frequency phase variation in clinical isolates [82], but the systems do have their distinctive differences. HmbR binds Hb and accepts haem as a monopartite system, whilst HpuAB is a bipartite system consisting of two proteins working in partnership HpuA, a lipoprotein attached to the outer membrane of the cell by its lipid anchor and HpuB, the 22-strand transmembrane β-barrel (**Figure 3B**).

The ability of pathogenic *Neisseria* to use haem as an iron source is an additional arrow in the quiver of their virulence. Although strains without the Hb receptors have been isolated from patients, comparisons of receptor presence and phase variation status in disease versus carriage isolates of *N. meningitidis*, led to the suggestion that expression of one of these receptors is important for systemic infection [83]. Therefore, although neither Hb binding receptor is essential for infection, the presence of one or the other does appear to contribute towards pathogenesis.

#### HpuAB

HpuAB is functionally distinct from HmbR due to its ability to acquire haem from the Hb:Hp complex. It is a system used not just by *Neisseria* pathogens but other pathogens from the wider *Neisseriaceae* family, including *Kingella kingae* and *Eikenella corrodens*. *K. kingae* is commonly found in the oropharynx of children and can lead to paediatric bacteraemia, osteomyelitis and septic arthritis [84]. *E. corrodens* is a normal part of the oral flora in children and adults, this asymptomatic colonisation can, however, lead to infections such as periodontitis, osteomyelitis, and endocarditis [85]. Although red blood cell lysis is an important step to haem acquisition from Hb, only *E. corrodens* and *K. kingae* of the named *Neisseriaceae* family pathogens have been described to be haemolytic [86, 87].

The *hpuA* gene is located directly upstream of *hpuB* and both genes are transcribed from an iron repressible promoter [88]. Although HpuB, as the TBDT, is seen as the main feature of the HpuAB system, both proteins are needed for efficient functionality (**Figure 3B**). A 2004 flow cytometry study into the functionality of each protein showed that HpuB expressed alone is able to bind Hb and Hb:Hp [89]. The study also showed that in the absence of HpuA, the binding capacity of the system was reduced and HpuB was less able to dissociate from Hb; speed of dissociation after nutrient extraction is an important factor in continual acquisition of haem from Hb. Wild type HpuAB was shown to dissociate from Hb very rapidly and efficiently. 50% of bound Hb vacates the binding site after addition of new Hb and that number increases to 80% for the dissociation of bound Hb:Hp. The study also reported that there was no observation of HpuA alone binding to Hb or the Hb:Hp complex through flow cytometry [89]. Just over a decade later, evidence of a direct HpuA-Hb interaction were presented: a HpuA homologue from *Kingella denitrificans* was shown to weakly bind Hb independently using pull down assays, isothermal titration calorimetry, nuclear magnetic resonance and crystallography. This HpuA shares 30% sequence identity and 48% similarity with its clinically relevant relative in *N. gonorrhoeae*. Due to this discovery, HpuA is secured in its description as an enigmatic, yet essential component of this iron acquisition system [90].

Structural analysis of HpuA revealed ‘a single domain comprising a C-terminal small β-barrel and an N-terminal loose β-sandwich'. The structure of the HpuA:Hb complex revealed some significant molecular details regarding its Hb binding capabilities: two functionally important loops involved in binding Hb (loops 1 and 5) interact with both the a and b chain of Hb, burying hydrophobic side chains and also forming several hydrogen bonds. However, these loops that are critical for substrate binding are not conserved across *Neisseriaceae* bacteria beyond the conservation of prominent hydrophobic residues [90].

Although the HpuAB system has been investigated for some time, the structure of HpuB has not yet been resolved. A model of the protein has been created using the crystal structure of ShuA (shares 16% identity with HpuB) as a template and this model outlines a β-barrel structure with an N-terminal plug domain [91]. The model is likely reliable when considering the transmembrane β-strands and the plug domain of the protein. However, as explained by Harrison *et al.* (2013), it becomes unreliable when focusing on the extracellular loops that are predicted to be involved in obtaining haem from Hb or the Hb:Hp complex. For example, loop 2 and 8 were too long to be modelled effectively and loop 9 was subject to high sequence variation [91].

#### HmbR

HmbR is a monopartite Hb-binding TBDT from *N. meningitidis* which was identified more than two decades ago but still has no published structure. Hb utilisation experiments using stains in which the *hpuB* gene was inactivated identified a poly G tract in the *hmbR* gene that is responsible for an ‘on/off' phase variation switch. Strains possessing either nine or twelve consecutive G residues were able to utilise Hb, whereas strains with any other number of consecutive G residues were not due to a resultant frameshift. The switching rates of ‘phase on' to ‘phase off' variation ranged from 7 x 10^-6^ to 2 x 10^-2^ in different serogroups [92].

More recently than the characterisation of phase variation in HmbR, a series of experiments identified functionally signification domains, which inevitably focused on the putative extracellular loops. Mutations in loop 2 and loop 3 affected Hb binding completely, whilst mutations in loop 6 and loop 7 did not eliminate Hb binding capability but did result in a failure to utilise Hb. Interestingly, although loop 7 was not identified to be essential for Hb binding, it is essential for Hb utilisation, theoretically due to a conserved Histidine residue and YRVP and NPNL motifs, which are found in all known Hb receptors [93]

### Haemophilus

There are several species of the *Haemophilus* genus known to cause infection in human. Of these pathogens, *Haemophilus ducreyi* and *Haemophilus influenzae* have been reported to use Hb as an iron source. *H. ducreyi* is a Gram negative obligate human pathogen and the causative agent of the sexually transmitted disease chancroid, characterised by genital ulceration and is thought to enter through breaks in the epidermis [94]. *H. ducreyi* is mainly found in developing countries but can also be found sporadically in the ‘global west'. A key comorbidity of infection is the 10 to 100-fold increase in Human Immunodeficiency Virus (HIV) transmission amongst *H. ducreyi* infected individuals [95]. The opportunistic human pathogen *H. influenzae* is nonmotile, facultatively anaerobic and its strains are broken down into two groups depending upon the absence or presence of a polysaccharide capsule [96]. *H. influenzae* is isolated predominantly from the respiratory tract of humans and is a normal part of the commensal flora in the nasopharynx [97]. *H. influenzae* infections mostly manifest in conditions like pneumonia but can also result in meningitis and systemic bloodstream infections. The encapsulated *H. influenzae* type b (Hib) has been the target of a widely used conjugate vaccine which has dramatically reduced the prevalence of community-acquired pneumonia. Indeed, most invasive *H. influenzae* infections are now caused by non-typable strains (NTHI) of the pathogen [98].

Like all Gram negative bacteria that use haem from Hb as an iron source, *H. ducreyi* also has a TBDT system specific for this purpose named HgbA, which has been demonstrated to have a vital role in the virulence of the pathogen. Unlike the Gram negative organisms from other genera described herein, *H. ducreyi* exhibits an obligate requirement for haem, at a minimum concentration of 38 µM [99]. The organism also has a second TBDT called TdhA, which facilitates the use of free haem [100]. Interestingly, in a human infection model, *H. ducreyi* was unable to establish an infection when HgbA was knocked out, suggesting that expression of TdhA alone is not sufficient for *H. ducreyi* virulence [101].

Due to the obligate requirement of haem by *H. ducreyi*, HgbA has been a target for successful immunisation studies against the pathogen. In a study of immunised pigs, no viable *H. ducreyi* cells were recovered following infection of HgbA-immunised pigs, whereas mock-immunised pigs were not protected from the infection. The study also showed that antibodies acquired from HgbA-immunised pigs were bactericidal and offered protection against *H. ducreyi* [102]. The swine model was chosen because of similarities in infection symptoms and also because a single experimental infection with *H. ducreyi* did not protect the animals from future infections, another shared feature with humans [103].

HgbA is modelled to be a 22-strand transmembrane β-barrel protein with eleven putative extracellular loops. *In vivo* loop deletion analysis of the protein revealed that deletion of sequences in loops 5 and 7 of HgbA rendered the protein unable to bind Hb. This elimination of Hb binding capability was not seen in deletion mutants of the other nine loops, an observation that was confirmed in two separate formats. Anti-HgbA IgG antibodies generated during the immunisation study discussed above showed show that loops 4, 5, and 7 of HgbA were immunogenic and surface exposed. Furthermore, anti-HgbA IgG specifically directed against loops 4 and 5 were shown to block Hb binding by *H. ducreyi* [104].

*H. influenzae* also cannot survive without an exogenous source of haem and different numbers and combinations of Hb- and Hb:Hp-binding TBDTs have been identified in different strains, for example HgbA, HgbB, HgbC in NTHI strain N182 and HhuA in NTHI strain TN106 [105, 106]. These proteins all share approximately 40-50% identity with each other and with HgbA of *H. ducreyi*. Deletion mutation experiments revealed that expression of any of HgbA, HgbB or HgbC allowed *H. influenzae* N182 to retain wild type level growth with Hb as the sole iron source [107]. Where the iron source was the Hb:Hp complex, mutants expressing only HgbA or HgbC grew significantly better than mutants expressing only HgbB. Interestingly, in a mutant where HgbA, HgbB and HgbC were deleted, NTHI N182 was still able to use Hb as an iron source to near wild type levels. This was in contrast to the significant impairment in the mutant's ability to utilise the Hb:Hp complex. A further *tonB* deletion mutation made to the *hgbA, hgbB, hgbC* triple mutant did strip it of its ability to grow using Hb as an iron source, indicating that there was another TBDT assisting in NTHI N182's ability to use Hb as an iron source. Proteomic analysis showed that HxuC, a TBDT previously characterised to be involved in the use of free haem and haemopexin [108] was supplementing the activity of the HgbA, HgbB and HgbC proteins. In a mutant where all four of the aforementioned TBDTs were deleted, NTHI N182 did eventually lose the ability to utilise Hb [107]. Although a HxuC deletion mutant was reported to lose the ability to utilise low concentrations of free haem *in vitro,* the mutation had no observable implication on the ability of *H. influenzae* to use Hb [108], this suggests a significant amount of redundancy employed by this organism for which haem is an essential nutrient.

### Pseudomonas aeruginosa

As with the Gram positive section, this is not an exhaustive list of Gram negative pathogens able to use Hb an iron source. It is also worth mentioning *Pseudomonas aeruginosa* as this Gram negative pathogen exhibits an interesting within-host adaptation towards iron acquisition from Hb. *P. aeruginosa* are small Gram negative rods that survive in many niches including in soil and aquatic environments, but also infect humans by colonising the respiratory tract and can also infect the urinary tract, skin and soft tissue [109]. *P. aeruginosa* is able to acquire iron using a variety of different means including secreting siderophores and haemophores. Because of its ability to infect via the respiratory tract, *P. aeruginosa* infections are prevalent in cystic fibrosis (CF) patients and can exhibit chronic long-term infections. Interestingly, *P. aeruginosa* undergoes genetic adaptation during the course of infection. In 2014, Lykke *et al.* reported that one of these genetic adaptations increases the ability of *P. aeruginosa* to acquire haem from Hb, coincidental with the loss of pyoverdine production, one of its most important iron chelating siderophores [110]. A mutation in the promoter for PhuR, a *P. aeruginosa* haem specific TBDT, conferred a significant growth advantage compared to the wild type in media in which Hb was the only iron source, an advantage not observed with other iron sources. This suggests a possible increase on reliance of haem over other iron sources during long term chronic infections but no direct binding of Hb has been observed.

### Acinetobacter baumannii

*Acinetobacter baumannii* is a non-motile, gram-negative coccobacillus bacterium, which accounts for most *Acinetobacter* infections in humans and can also infect other mammals. Symptomatic human infections can be multifaceted both in infection site and severity. Infections can start in the blood, urinary tract, lungs or in open wounds around the body. *A. baumannii* is a robust pathogen, able to survive on a variety of surfaces and equipped with a genome that allows for resistance to last line antibiotic treatments [111]. Indeed, *A. baumannii* is now at the top of the World Health Organisation's list of superbugs in need of urgent development into alternative therapeutic treatments [112]. Aside from genomic resistance to antibiotics, *A. baumannii* is also known to demonstrate desiccation resistance, biofilm formation and notably pairs these environmental persistence features with virulence factors such as secretion systems and surface glycoconjugates. [111]

The story of *A. baumannii* infection and nutritional requirement is a familiar one: the need for crucial nutrients, like iron, has resulted in sophisticated and effective mechanisms of nutrient acquisition from the host itself. Iron acquisition via the capture of haem has been suggested as a major virulence factor in *A. baumannii* [113]. Like other Gram negative bacteria seeking to import haem, *A. baumannii* utilises TBDTs to overcome the barrier of its double membrane cell wall. Two *A. baumannii* TBDTs have been observed to function as haem transporters, firstly the ubiquitous HemTR, which appears sufficient for the bacteria to survive when abundant haem is the sole iron source, and secondly, HphR, which is necessary for growth on Hb as an iron source [114]. HphR is found on a gene cluster known as the *hemO* locus alongside other proteins functionally relevant to the piracy of haem from Hb in *A. baumannii.* This review has previously described both monopartite and bipartite TBDT systems, where the TBDT itself is reported to both bind Hb and accept its haem unilaterally or with the help of an extracellular anchored lipoprotein partner respectfully. The HphR system of *A. baumannii* is a different type of bipartite system. Downstream of the gene for *hphR* on the *hemO* locus is a gene encoding HphA [114]. HphA is a secreted protein that has been reported to bind Hb (**Figure 3C**) through pull down experiments where HphA was pulled down by Hb conjugated agarose resin and Hb was shown to bind to Apo HphA-GST immobilised to glutathione beads [114]. In contrast to lipoprotein HpuA of *Neisseria*, which is only apparently necessary for the high affinity interaction between cells and Hb, HphA also binds to haem directly. This leads to a model where HphA is the initial Hb and haem receptor, which then passes the haem onto the TBDT HphR for import. Whether HphA is the sole protein of the system that can bind to Hb or whether HphR is also involved in Hb-binding remains to be tested.

It has been demonstrated that haem uptake via proteins encoded on the *hemO* locus are important for virulence and systemic spread with mouse infection models demonstrating HphR mutants had the worst survival rates of *hemO* protein mutants [114]. Interesting, although HphA is the protein demonstrated to have Hb binding capabilities, it is reported to be an accessory to enhancing infection, while HphR is essential to virulence. This suggests that the essential transport of haem can occur without the Hb-binding haemophore, in which case the haem may come from other sources, albeit these would not be in such abundant quantities as Hb.

## USE OF HAEMOGLOBIN BY MYCOBACTERIA

Bacteria of the *Mycobacterium* genus have a unique cell surface structure that does not neatly fit into either the Gram positive or Gram negative categories. *Mycobacterium tuberculosis* can infect any tissue within a human host but often infects the respiratory system and causes the disease tuberculosis.

Access to iron is vital for *M. tuberculosis* virulence and as a result, the bacteria have evolved multiple systems to meet their needs. Iron chelating siderophores called mycobactins are produced by *M. tuberculosis* and are essential for growth in macrophages, where mycobactin synthesis genes are upregulated [115, 116]. Siderophore use notwithstanding, the iron acquiring prowess of *M. tuberculosis* may extend to the direct utilisation of host iron and haem carrying proteins too. Indeed, the ability to use haem iron was confirmed by experiments in which iron uptake deficient mutants were created by disruption of mycobactin/exomycobactin biosynthesis. These mutants were grown in several iron specific environments, including those where Hb was the sole iron source [117]. The amount of iron available has been linked to likelihood of infection, with iron overload reported to increase the risk of tuberculosis, of tuberculosis treatment failure, and of mortality in tuberculosis patients [118]. At the opposite side of the iron availability spectrum, it has also been demonstrated that extended periods in an iron-deprived environment results in *M. tuberculosis* entering a persistent state, a likely characteristic of chronic tuberculosis [119].

**Figure 4:**
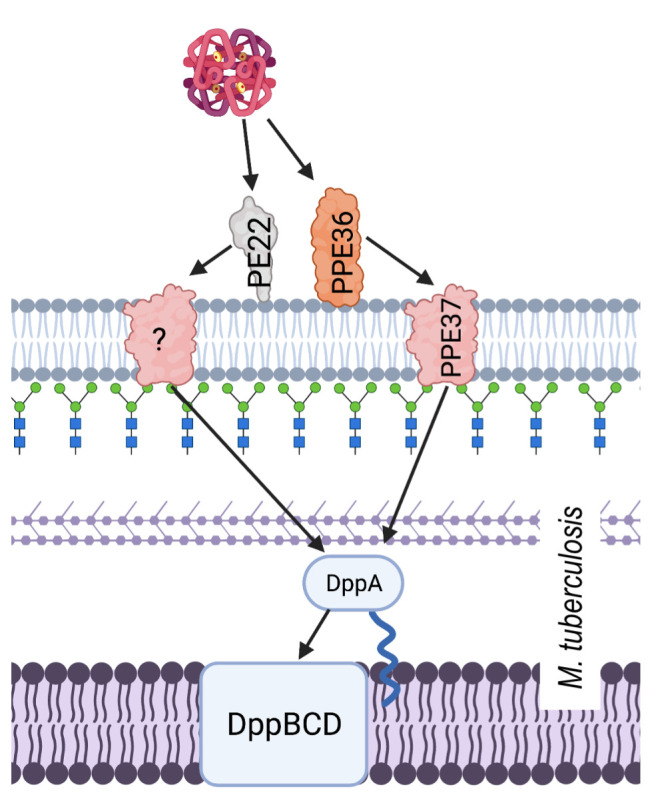
FIGURE 4: A schematic showing haem acquisition from Hb by *M. tuberculosis*. The cell surface proteins PPE36, PPE22 and PPE62 are able to bind haem, facilitating the use of Hb. The haem is transported into the periplasm by PPE36 or another functionally similar but unknown protein. Once in the periplasm the haem is bound by lipoprotein DppA, and transported into the cytoplasm by ABC transporter system DppBCD.

Hb utilisation in *M. tuberculosis* involves surface exposed proteins, which are suspected to bind Hb, and a haem specific ABC transporter system. Hb binding by *M. tuberculosis* is facilitated by membrane proteins PPE36/PE22 and PPE62. PPE36/PE22 and PPE62 are separate systems that are both capable of binding haem and facilitating acquisition from Hb at the cell surface in order to make haem available for transport into the cytoplasm by the Dpp ABC transporter system (**Figure 4**) [120]. Although the mechanism by which haem is obtained from Hb is still unknown, largely due to the lack of published structures, we do know that PPE36 is the most essential of the surface exposed protein systems. Deletion mutant experiments showed that ΔPP36 mutants were completely halted in their growth with haem as a sole iron source, whereas ΔPP62 mutant growth was only partially impaired [121]. Another protein, PPE37, is also contested to play a role in haem acquisition by *M. tuberculosis*. There have been no experiments to suggest that PPE37 is directly involved in Hb binding and even its role in haem utilisation is disputed. Exploiting the fact that *Mycobacterium bovis* is unable to acquire haem iron, Tullius *et al.* (2019) introduced the *ppe37* gene to *M. bovis* and found that the resulting mutant was able to acquire haem iron in similar levels as *M. tuberculosis.* In addition to this, Tullius *et el*. reported that an N terminal deletion in PPE37 was responsible for the loss of the phenotype in wild type *M. bovis* and that *M. tuberculosis* strains that also possess the altered PPE37 protein (up to 60% of sequenced genomes) would be equally incapable of haem acquisition [122]. By contrast, Mitra *et al.* (2019) found that deletion of PPE37 in the avirulent *M. tuberculosis* strain mc^2^6206 had no effect on growth and that this mutant grew on minimal media in the presence of 10 µM hemin or 2.5 µM human Hb identical to that of the wild type [121]. A consensus on the mechanism of haem uptake from Hb in *M. tuberculosis* therefore remains elusive.

The haem-specific ABC like transporter of *M. tuberculosis* is named Dpp and is made up of four proteins DppA, DppB, DppC and DppD. By infecting macrophages with different iron deficient mutants including a Δ*dpp* mutant, Mitra *et al.* (2019) were able to demonstrate the importance of this system for *in vivo M. tuberculosis* survival. Of the proteins in this system, DppA, specifically through its arginine 179, possesses the substrate specific binding ability that makes it essential for Hb free haem utilisation by the entire cell.

## DISCUSSION

This review has focused on a wide range of pathogenic bacteria with one thing in common, the ability to use haem from Hb as an iron source through cell surface piracy, using a variety of reported Hb binding proteins (**Table 2**). Above we have described the information available about the systems they employ to this end. The more open questions on this topic concern how actively these systems engage with Hb itself, the availability of Hb for these pathogens, how important this iron source is for infection and whether the systems can be targeted therapeutically.

**TABLE 2.** **Haemoglobin binding proteins in human pathogens.** Human pathogens that use Hb as an iron source usually bind Hb through specific surface exposed membrane proteins, extracellular lipoproteins or secreted haemophores. Pathogens can have more than one Hb binding system and these proteins can have single or multiple substrates.

**Hb binding organism**	**Hb binding protein**	**Functional domains**	**Host Haemoprotein Substrate**
*Corynebacterium diphtheriae*	HtaA [33]	CR domains	Hb, Hb:Hp, Myoglobin
*Corynebacterium diphtheriae*	ChtaA/ChtC [36]	CR domains	Hb:Hp
*Corynebacterium diphtheriae*	HbpA [37]	CR domains	Hb:Hp
*Staphylococcus aureus*	IsdB [45]	NEAT domains	Hb
*Staphylococcus aureus*	IsdH/HarA. [44]	NEAT domains	Hb, Hb:Hp
*Streptococcus pyogenes*	Shr *[50]*	HID & NEAT domains	Hb, Hb:Hp, Myoglobin
*Bacillus anthracis*	IsdX1/X2 [56]	NEAT domains	Hb
*Bacillus anthracis*	Hal *[58]*	NEAT domains	Hb
*Listeria monocytogenes*	Hbp2 [63]	NEAT domains	Hb
*Clostridium perfringens*	ChtD/ChtE *[66]*	NEAT domains	Hb
*Shigella dysenteriae*	ShuA [74]	TBDT	Hb
Pathogenic *Neisseria*	HmbR [93]	TBDT	Hb
Pathogenic *Neisseria*	HpuA [90]	Lipoprotein	Hb, Hb:Hp
Pathogenic *Neisseria*	HpuB [89]	TBDT	Hb, Hb:Hp
*Haemophilus ducreyi and Haemophilus influenzae*	HgbA [101]	TBDT	Hb
*Haemophilus influenzae*	HgbB & HbgC [105]	TBDT	Hb, Hb:Hp
*Haemophilus influenzae*	HhuA [106]	TBDT	Hb, Hb:Hp
*Pseudomonas aeruginosa*	PhuR [110]	TBDT	Hb
*Acinetobacter baumannii*	HphA [114]	Lipoprotein	Hb
*Mycobacterium tuberculosis*	PPE36/PE22 [120]	PPE domain	Hb
*Mycobacterium tuberculosis*	PPE62 [120]	PPE domain	Hb

### Intimacy of interactions with Hb

Throughout, we have highlighted the experimental techniques used to determine the ability of haem acquisition systems to bind directly to Hb as their substrate, and the reliability of some of these techniques have now been called into question.

For some of the Gram positive pathogens mentioned here, the binding of their surface exposed proteins to Hb is facilitated by NEAT domains, often more than one. Specific motifs have been identified within NEAT domains that are indicative of either haem (S/YXXXY) or Hb (F/Y)YH(Y/F) binding capabilities [44]. While some NEAT domains have been proposed to bind both haem and Hb, the lack of domains containing both motifs makes this unlikely. NEAT domain proteins identified as capable of binding both haem and Hb possess distinct NEAT domains for binding each substrate. Experimentally, haem binding of NEAT domains is well evidenced by solved structures of NEAT domains bound to haem and UV-vis spectroscopy. For Hb binding, ELISA experiments have been the main demonstrative tool. *C. diphtheriae* proteins HtaA and ChtB [34] [36], *B. anthracis* proteins IsdX1/X2 [57] and *L. monocytogenes* protein Hbp2 [63] are examples ELISA demonstrated Hb binding but follow up NMR experiments aimed at detecting haem binding directly failed to detect an Hb interaction for *B. anthracis* IsdX1/X2 [123]. One plausible explanation for this discrepancy is that in most ELISA experiments, the Hb used, usually commercially bought, contains a variety of breakdown products including multiple oligomeric and oxidation states and that immobilisation of these products with more readily accessible haem produces false positive results. It remains to be tested if the Hb- and HbHp-binding of *C. diphtheriae* proteins HtaA and ChtB observed in ELISA experiments can be validated using a different technique.

In addition to this, there must be clarity when describing the ability of aforementioned Hb binding proteins to actually engage in the actively stripping of haem from Hb. Do they engage in active stripping or just bind to Hb in a well-placed position to accept haem when spontaneously released? It is tempting to speculate an active removal of haem from Hb as the IsdB and IsdH proteins of *S. aureus* have been shown to distort the Hb structure to weaken its binding to haem [46, 124]. Similarly, although not directly relevant to Hb, the TonB dependent receptors HasR and TbpB have also been show to actively dissociate their substrates from their respective target: haem from the haemophore HasA and iron from transferrin [76, 125]. However, we note that an active role of haem removal from Hb has not been demonstrated for the CR domains, TonB dependent receptors or *Mycobacteria* proteins.

### Hb availability

The majority of pathogens covered in this review are capable of causing systemic infections where they will encounter free Hb from spontaneous haemolysis. Most are also known to be haemolytic and the lysis of erythrocytes would further enhance their supply of the most abundant source of iron in the body, for which they have just the right tools to plunder. Indeed, of the pathogens listed in this review, only the organisms from the *Neisseria* and *Bacillus* genera are non-haemolytic on blood agar. However, for most of these pathogens, their usual modes of transmission involve host niches that one does not usually associate with an abundance of Hb and the selective pressure that has led to the evolution of Hb piracy systems may not be immediately obvious. The most common infection and transmission methods of these pathogens can be divided into three categories: those that involve surfaces of the respiratory system: *C. diphtheriae, S. pyogenes, S. aureus, N. meningitidis, M. tuberculosis, P. aeruginosa*, those that transmit via mucosal surfaces of genitalia: *N. gonorrhoeae, H. ducreyi,* and those that transmit via the faecal-oral route: *C. perfringens, S. dysenteriae, L. monocytogenes*. Although the concentrations of Hb encountered by these bacteria through their infection and transmission cycles is not known, these mucosal locations are not normally associated with an abundance of Hb-containing erythrocytes.

Concentrations of Hb in the oral, nasal and pharyngeal cavities that act as a niche for *S. aureus, S. pyogenes, C. diphtheriae* and *N. meningitidis* have not been directly defined, but investigations into salivary Hb as a potential diagnostic tool for periodontitis have identified an average of 6 µg/mL of Hb in saliva of healthy individuals [126]. Whether this level is indicative of the concentrations available for these organisms in their more intimate associations with the mucosal surfaces and whether it is sufficient to support growth remains to be demonstrated. For *M. tuberculosis* and *H. influenzae* that infect deeper respiratory tissue, speculation as to the source of their targeted Hb may extend beyond erythrocytes. Although originally understood to be expressed solely by erythrocytes, expression of Hb has been detected both by activated mouse macrophages and by alveolar epithelial cells [1, 127, 128]. Whether either of these cell types could be a source of Hb for *M. tuberculosis*, whose pathogenesis is explicitly linked with alveolar macrophages, has not been directly investigated, but a *dpp* knockout strain of *M. tuberculosis* was impaired for survival in macrophages, suggesting that haem is an important iron source in this niche [121]

The urogenital mucosa that support growth of *N. gonorrhoeae* and *H. ducreyi* are also not often associated with erythrocytes and Hb. Are Hb receptors important during infection of these surfaces, or only for when these organisms spread systemically? In the case of *N. gonorrhoeae*, the dominance of iron acquisition using transferrin receptors is well documented [129]. Notwithstanding, there is evidence that Hb receptors are employed to benefit from the increased blood availability during menses. Hb receptor ‘on' strains were far more likely to be isolated from female patients than from male patients and the receptor is expressed significantly more often by isolates from the earlier stages of the menstrual cycle [130]. In chancroid disease caused by *H. ducreyi* infection, biopsies of resulting genital ulcers have revealed a base layer of superficial necrosis that includes fibrin, leukocytes and erythrocytes, lysis of which could yield Hb as an iron source [131].

The tissue damage that results from *S. dysenteriae* infection often leads to diarrhoea containing blood and this is a potential source of haem for these organisms during their infection cycle. The other gastrointestinal pathogens listed briefly above are less commonly associated with dysentery and for these we can only speculate that the sources of Hb could be dietary during the infection cycle, or that the Hb receptors are employed only during systemic infection.

### Hb receptors in virulence and therapeutic intervention

The ability of pathogens to circumvent host nutritional immunity and to exploit the host proteins involved has long been touted as a possible focus for therapeutic intervention. With respect to therapeutic potential, the most significant question in the case of the systems described above is whether haem acquisition from Hb makes up a large enough proportion of the total iron needs of the pathogen to constitute a worthwhile target. The answer to this question differs between bacteria, and the universal ability to bind Hb does not indicate that Hb-binding systems will be a universally expedient target. For Hb receptors that are critical to the survival of a bacterial cell, direct inhibition of the system could be a useful avenue to explore. Alternatively, as these are surface exposed proteins, their utility as vaccine antigens has potential.

The degree to which the pathogens featured here are reliant on haem for iron is variable. The elimination of haem piracy systems restricts bacterial growth to varying degrees, and in some cases abolishes pathogenicity or even proves lethal. Obligate human pathogen and haem utiliser *H. dureyi* requires a minimum haemin concentration of 38 µM for growth and, whilst it is able to pirate this haem from Hb using Hb receptor HgbA, it also possesses TdhA, a free haem receptor that allows it to utilise free haem [99]. However, haem acquisition via TdhA is not sufficient to support infection, as demonstrated by a human model of infection where pustules did not develop in adults infected with HgbA-knockout *H. dureyi* [101]. This requirement for haem iron, combined with the lack of other iron sources, means that the *H. ducreyi* Hb receptor HgbA is a promising candidate for a vaccine antigen [104]. For *H. influenzae*, the redundancy of different Hb receptors adds an extra layer of complexity to any attempt of therapeutic intervention into this process.

In the *S. aureus* Isd system, both IsdB and IsdH bind directly to Hb [132] [42] and deletion of *isdB* resulted in reduced virulence in a mouse model [48]. Interestingly, deletion of *isdH* did not result in reduced virulence in this model, but the results from *isdB* deletion demonstrate the importance of Hb utilisation in virulence. Interestingly, *S. aureus* has also been shown to have a preference for haem iron [133]. Starving colonies of iron prior to growing them in a culture with equal amounts of labelled haemin and transferrin led to a much greater depletion of haemin than transferrin. There is no evidence yet to argue that the Hb binding proteins of *S. aureus* are immunogenic but the fact that the Hb piracy system plays a role in virulence suggests that targeting this system would reduce its virulence.

A study of *M. tuberculosis* persistence within macrophages where genes involved in various iron acquisition systems were knocked out found that mutants with deletions of proteins in the Dpp system had the worst survival rates [121]. The importance of macrophage survival in TB infection positions the Dpp system, particularly DppA as a potential therapeutic target for intervention to block haem uptake through this ABC transporter. Further research is needed into the surface proteins that may bind directly to Hb, in order to reveal whether these also constitute worthwhile targets.

Hb receptors are also crucial for full infection in other pathogens featured here. Similar to the *M. tuberculosis* example above, DppA orthologue SiaA of *S. pyogenes* has also been demonstrated to play a key role in virulence. In a mouse model of *S. pyogenes* infection, the deletion of the haem and Hb binding protein SiaA resulted in significantly increased host survival rate, reduced skin lesion size, and reduced systemic dissemination [54]. Similarly, in *C. perfringens*, the double deletion of proteins proposed to be responsible for Hb binding (ChtD and ChtE) resulted in reduced virulence in mouse myonecrosis models [66].

The pathogens featured in this review are all able to bind Hb and accept its haem, thereby serving as examples of the potential exploitation of Hb: the largest source of haem and the largest iron reservoir in the human body. Whilst not all of these pathogens have a proven reliance on this iron source for virulence or even survival, and indeed, none of them have an obligate requirement for Hb when grown outside the host, it is reasonable to speculate that pathogenic organisms with an obligate requirement for haem are to a high degree reliant on Hb availability and that other pathogens with receptors for Hb employ these for their advantage. The wide range of bacteria that do express Hb piracy systems and the evidence that for some at least this is important for *in vivo* growth, does raise intriguing mechanistic questions about how piracy is accomplished and evolutionary questions about the pressures that have led to the systems developing. These questions are intriguing in their own right, even when severed from the potential for therapeutic targeting.

## Abbreviations:

ABC: – ATP binding cassette,
Hb: – haemoglobin,
HID: – Hb interacting domain,
Hp: – haptoglobin,
met-Hb: – metalloprotein form of Hb,
oxy-Hb: – Hb bound to oxygen,
TBDT: – TonB-dependent transporter.
